# Molecular docking and pharmacokinetic evaluation of natural compounds as targeted inhibitors against Crz1 protein in Rhizoctonia solani

**DOI:** 10.6026/97320630017660

**Published:** 2021-06-30

**Authors:** Arshi Malik, Sarah Afaq, Basiouny El-Gamal, Mohamed Abd Ellatif, Waleed N Hassan, Ayed Dera, Rana Noor, Mohammed Tarique

**Affiliations:** 1 Department of Clinical Biochemistry, College of Medicine, King Khalid University, Abha, Saudi Arabia; 2 Center for Interdisciplinary Research in Basic Sciences, Jamia Millia Islamia, Jamia Nagar, New Delhi-110025, India; 3 Department of Medical Biochemistry, Faculty of Medicine, Mansoura University, Mansoura, Egypt; 4 Departments of Clinical Laboratory Science, College of Applied Medical Science, King Khalid University, Abha, Saudi Arabia; 5 Department of Biochemistry, Faculty of Dentistry, Jamia Millia Islamia, New Delhi110025, India

The first author in "Molecular docking and pharmacokinetic evaluation of natural compounds as targeted inhibitors against Crz1 protein in *Rhizoctonia solani* by Malik *et al.*(2019) should be Arshi Malik (2019) Bioinformation. 2019 15:227. PMID: 31285645

